# A web-based interactive framework to assist in the prioritization of disease candidate genes in whole-exome sequencing studies

**DOI:** 10.1093/nar/gku407

**Published:** 2014-05-06

**Authors:** Alejandro Alemán, Francisco Garcia-Garcia, Francisco Salavert, Ignacio Medina, Joaquín Dopazo

**Affiliations:** 1 Computational Genomics Department, Centro de Investigación Príncipe Felipe (CIPF), Valencia 46012, Spain; 2 Bioinformatics of Rare Diseases (BIER), CIBER de Enfermedades Raras (CIBERER), Valencia 46010, Spain; 3 Functional Genomics Node, (INB) at CIPF, Valencia 46012, Spain

## Abstract

Whole-exome sequencing has become a fundamental tool for the discovery of disease-related genes of familial diseases and the identification of somatic driver variants in cancer. However, finding the causal mutation among the enormous background of individual variability in a small number of samples is still a big challenge. Here we describe a web-based tool, BiERapp, which efficiently helps in the identification of causative variants in family and sporadic genetic diseases. The program reads lists of predicted variants (nucleotide substitutions and indels) in affected individuals or tumor samples and controls. In family studies, different modes of inheritance can easily be defined to filter out variants that do not segregate with the disease along the family. Moreover, BiERapp integrates additional information such as allelic frequencies in the general population and the most popular damaging scores to further narrow down the number of putative variants in successive filtering steps. BiERapp provides an interactive and user-friendly interface that implements the filtering strategy used in the context of a large-scale genomic project carried out by the Spanish Network for Research in Rare Diseases (CIBERER) in which more than 800 exomes have been analyzed. BiERapp is freely available at: http://bierapp.babelomics.org/

## INTRODUCTION

Recent advances in high-throughput sequencing technologies have made it possible to sequence whole genomes or exomes at unprecedented speeds and low costs. In particular, targeted sequencing of exomes has been extensively and successfully used to discover disease genes in Mendelian disorders (1,2) or in cancer (3,4). However, with more than 30 000 variants found per exome (1), finding disease-causing genes is a cumbersome, time-consuming task that often requires intensive human intervention (5). 

In spite of the obvious need for tools that facilitate the gene prioritization process, there are no many open solutions currently available (6). Most of the available tools cover the primary analysis (QC, alignment and variant calling) (7–9) that ends up in a list of variants found in sequencing experiments (VCF file) that can be annotated with different programs, such as VARIANT (10), ANNOVAR (11), etc.

In the case of inherited diseases or *de novo* syndromes, the availability of sequencing data of parents, siblings or close relatives can significantly help in the process of finding candidate disease genes. Actually, more sophisticated tools can use such information to help in the detection of disease-causing variants segregating along family pedigrees (12,13) or somatic mutations in cancer (14). These tools increase the precision of the calling process but lack, in some cases, user-friendliness and fail to provide other filtering steps. The only tool that enables more filtering steps, KGGSeq (15), is a command line application.

BiERapp fills the gap that leads from the list of predicted variants (VCF file) to the final candidate disease-gene list by providing an interactive, web-based, easy-to-use framework. The tool allows for the consecutive application of filters that include segregation in familial cases (with different inheritance modes that can be easily defined), allelic frequencies in the general population, mutational consequences and other that narrow down the number of putative variants to a small number of promising candidate variants.

## MATERIALS AND METHODS

### Input

BiERapp accepts standard VCF formats (16), typically a multi-sample VCF corresponding to several samples of one or several families. Sporadic patients or case-control studies can also be analyzed within the same framework.

**Figure 1.**
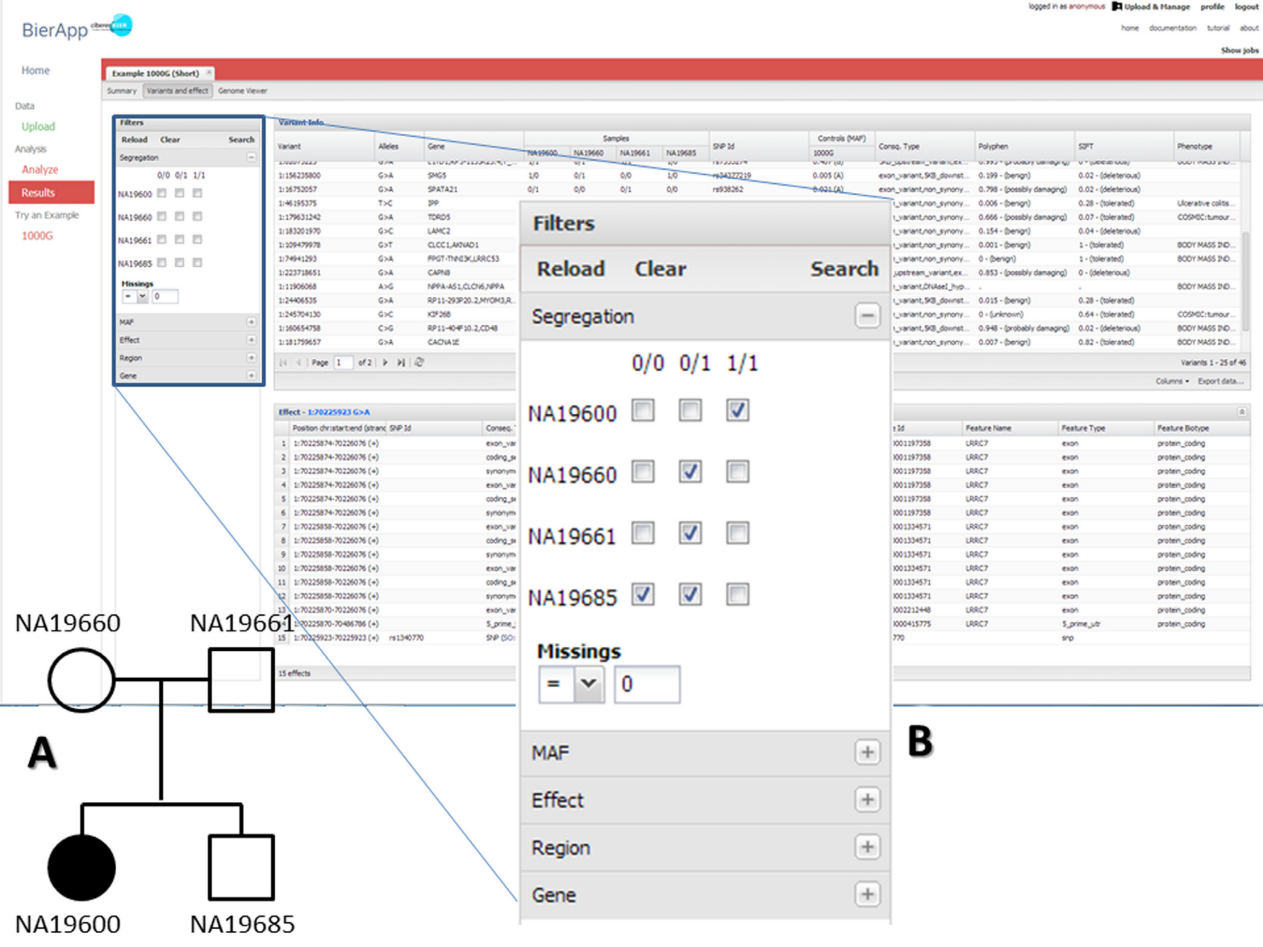
The main screen of BiERapp and the filtering panels. (**A**) Example of family pedigree with an affected descendant (NA19600), two carrier parents and an unaffected descendant. (**B**) The segregation filter that allows easily defining any inheritance mode (see the text for an explanation).

The ‘Data Upload’ entry of the left menu of the main screen (see Figure 1) allows uploading the data from the user's local disk. Using the ‘Upload local file’ button, the user can browse their local disk and load the VCF file. In the dialog window that pops up, the user has some options to identify the data and to associate some information with them. The selected local file is uploaded to the server, where several VCF files can be stored. Pressing the run button the data set goes to the ‘jobs’ panel, on the right, where it is indexed. This process may take several minutes, depending on the data size. For example, a VCF file containing some 40 000 variants may take about 10 min. Supplementary Figure S1 shows a linear relationship between indexing runtimes and the number of variants, with different slopes for different number of exomes. The figure shows a representation of indexing runtimes for different numbers of exomes (from 1 to 20) and different numbers of variants (up to 100 000). Once the job is finished, the user can click on it and the prioritization by applying successive filtering steps can start. Several jobs can independently be run and invoked for analysis.

### Data sources

The information used for the annotation of the position is stored in CellBase (17), which collects it from different sources. The identifiers of single nucleotide polymorphism (SNP) are extracted from dbSNP (18). The consequence types of the variants and their predicted pathologic effect, according Polyphen (19) or SIFT (20) indexes, are extracted from Ensembl (21). The Minor Allele Frequencies (MAFs) of the variants are calculated for the populations derived from the 1000 genomes (22) and Exome Sequencing Project (ESP) (23) studies. In the case of 1000 genomes, the VCF files are downloaded from the server (http://www.1000genomes.org/ftpsearch) and the proportions of the different genotypes are calculated and the allelic frequencies derived from them. In the ESP, the genotype counts were directly available in the server (http://evs.gs.washington.edu/EVS/) and were used to derive MAFs. The disease phenotype from HGMD (24), ClinVar (25) and UNIPROT (26) databases is taken from Ensembl as well (21).

### Prioritization of variants by successive filtering

Each prioritization (‘job’) has three associated screens that facilitate the filtering steps. The first one, the ‘Summary’ tab, displays a statistic of the data set analyzed, containing the samples analyzed, the number and types of variants found and its distribution according to consequence types. The second screen, in the ‘Variants and effect’ tab, is the actual filtering tool, and the third one, the ‘Genome view’ tab, offers a representation of the selected variants within the genomic context provided by an embedded version of the Genome Maps tool (27).

The prioritization of variants is conducted by means of a consecutive filtering strategy in which different filters are applied to reduce the number of potential disease variants. The filters that can be applied are the following.

- *Segregation filter*: This filter allows specifying the distribution of alleles that are compatible with the pedigree analyzed, given the inheritance model of the disease. Figure 1A shows an example with a very simple family ‘pedigree’. If the disease is autosomal recessive then: both parents must be carriers and have the alleles in a configuration 0/1 (meaning that one of the alleles is the reference allele and the other one is the alternative allele), the affected individual must have an allelic configuration of 1/1 (that is, homozygote for the alternative allele, which is the potential causative agent) and the unaffected individual could either be 1/0 or 0/0 (that is, heterozygote or homozygote for the reference allele, respectively). Any pedigree and inheritance model, including incomplete penetrance, can be specified in the interface (Figure 1B) using very simple rules in an intuitive manner. Obviously, the application of the filter discards Mendelian inconsistencies.
- The segregation filter can also be used to analyze case-control experimental designs by setting the configuration of alleles in a way that differentiates among them (e.g. 1/1 for cases versus 1/0 or 0/0 for controls in the case of a mutation that causes loss of function).
- Since sequencing errors can happen, or some of the cases could have a different mutation, the filter can accept a certain degree of uncertainty in the application of the filters. Thus, the data available for some samples could be missing for the filtered position. This maximum number of missing values accepted can be indicated in the corresponding box (Figure 1B). When *N* missing values are accepted, the positions that either fulfill the zygosity criteria or are missing are first collected. Then only those positions having *N* or fewer samples with missing values are displayed.
- *Consequence type filter*: As a first option, variants with a predicted severe effect are the best candidates. Therefore, ‘non-synonymous’ and ‘stop lost’ are the initial preferred consequence types.
- *Allelic frequency filter*: This is another quite useful filter that can be used to discard variants with a relatively high MAF in the population. These variants are unlikely to be causative of many hereditary disorders. MAFs are obtained from the 1000 genomes (22) and ESP (23) projects.
- *Regions filter*: Should previous information on regions associated with the disease be available from linkage disequilibrium studies, BiERapp allows focusing on these particular regions, which can be specified in the corresponding box.
- *Genes filter*: If some genes are of special interest, the analysis can be focused on them by specifying their names in the corresponding box.

When the filter is applied, a selection of filtered variants appears in the ‘Variant info’ panel. Each line corresponds to a variant for which the following information is displayed: (i) genomic position (chromosome:position); (ii) allelic change (reference allele > alternative allele); (iii) the name of the gene affected by the variant; (iv) the allelic composition of each sample analyzed (0/0, 0/1 and 1/1 for the homozygote reference allele, the heterozygote and the homozygote alternative allele, respectively; ./. accounts for low quality or low coverage positions); (v) the SNP identifier in dbSNP (18) if the variant is an already known SNP; (vi) the MAF in the population derived from the 1000 genomes (22) and ESP (23) studies; (vii) the consequence type of the variant; (viii) the predicted pathologic effect of the variants according to Polyphen (19) or (ix) SIFT (20) indexes (if the variant has more than one pathologic consequence, the most deleterious value of the indexes is displayed here); and the phenotype extracted from CellBase (17) as annotated in HGMD (24), ClinVar (25) and UNIPROT (26). Columns can be customized by hiding or rearranging them by directly clicking on them or using the button ‘columns’ in the lower right corner of the panel.

A second panel displays the effect that the variant selected in the first panel has over the different genomic features in which it is located (that can be more than one). It often occurs that the same variant affects different transcripts, with different effects, and also to other elements such as regulatory motifs, splicing motifs, etc. This panel provides exhaustive information on all the aspects of the possible effect of the variant and includes the following data: chromosomal position, SNP ID (if any), consequence type, amino acid change (if the feature is an exon and applies), the Ensembl gene name, the transcript ID (including a link to the Ensembl), the feature ID, the feature name, type and biotype.

If no variants compatible with the disease are found or further validations demonstrate that none of the selected variants were associated with the disease then the filters can be relaxed to increase the number of possible candidates. New prioritizations can be interactively generated by changing the filters and pressing the ‘search’ button in the ‘Variants and effect’ tab.

Finally, a third tab displays the selected variant within the genomic context using the Genome Maps (27) genome viewer, which is embedded in the application. Genome Maps provides a contextual view of the variant position within the genome, highlighting all the relevant features around (transcripts, genes, SNPs, etc.).

Filtering sessions are deleted once the web page is closed. Alternatively, there is a possibility of registering and logging in on a private session. In this case, all the analyses done are kept in a user's account in the server.

### Output

Data can be exported in comma delimited CSV format, which can be imported by any spreadsheet. In the ‘Variants and effect’ tab, the ‘variant info’ panel has in its lower right corner a button to export data. When clicking on it, a window pops up where the user can select the columns to save and the CSV file can be downloaded to the local disk.

### Technical details

BiERapp is an open source tool based on HTML5 and JavaScript. The application user interface has been developed in javascript with the Ext JS and the Bootstrap framework. BiERapp uses a fast and optimized indexing and annotating system based in SQlite for queries. All the filtering operations are carried out locally, in the user's browser. The relevant information on genes, variants, features, etc. used for the prioritization is remotely stored (and kept updated) in CellBase (17) and is provided through highly efficient web services.

In order to scale up and improve the database performance, a second implementation has been developed using MongoDB database. MongoDB is a distributed and scalable high-performance database. This implementation shows a much higher runtime performance and can scale up to Terabytes of data. BiERapp fetches data through Java RESTful web services which can query these two possible database implementations, making transparent for the user how the application is storing the data.

BiERapp makes an intensive use of new web technologies and standards, therefore only new browsers are fully supported. These include: Chrome 14+, Firefox 7+, Safari 5+, Internet Explorer 11+ and Opera 11+. Older browser like Chrome13−, Firefox 5− or Internet Explorer 9 may yield errors. Internet Explorer 6 and 7 are no supported.

## DISCUSSION

BiERapp is an extensively tested tool, which has been used during the last year by the BiER team (Bioinformatics for Rare Diseases Team; http://www.ciberer.es/bier). More than 800 exomes of patients of more than 70 different inherited pathologies, produced by the Spanish Network for Research in Rare Diseases (CIBERER, http://www.ciberer.es) and the Medical Genome Project (MGP; http://www.medicalgenomeproject.com), have been analyzed using BiERapp. Recent publications include the discovery of two new mutations in the BCKDK gene, responsible of a neurobehavioral deficit in pediatric patients (28), new mutations in different genes causing inherited retinal dystrophies (29–32) and metabolic diseases (33).

There are several programs available in which family information is used to improve the variant calling process like FamSeq (12), PolyMutt (13) or the recently published VariantMaster (34) which claim to be quite sensitive and especially devised to detect *de novo* variants. Other tools are also sophisticated variant callers specific for finding somatic variation in cancer (14). KGGSeq (15) is a java application with a filtering philosophy similar to those in BiERapp. However, the inheritance pattern filter seems to be too rigid and offers only a limited number of scenarios. None of the mentioned applications is a web server.

Apart from free applications there are several commercial solutions available. Ingenuity's Variant Analysis (http://www.ingenuity.com/products/variant-analysis) and Golden Helix's SNP & Variation Suite (http://www.goldenhelix.com/SNP_Variation/DNA-Seq_Analysis_Package/index.html) offer a sequential filtering strategy similar to BiERapp. The second one is a stand-alone application that offers a filtering step similar to what we have implemented in BiERApp.

The proposed web-based interactive framework has great potential to detect disease-related variants in familial diseases as demonstrated by its successful use in the CIBERER and MGP initiatives above mentioned. To our knowledge, BiERapp is the first free web tool that provides the possibility of applying a consecutive filtering approach to variants coming from a whole-exome sequencing study with this level of interactivity. The program manages Mendelian inheritance modes by providing an intuitive filter that allows reproducing any familial pedigree with any inheritance model and allows selecting variants (and genes with deleterious variants) that segregate with the disease in the family. The use of the filters is interactive and the results are almost instantaneously displayed in a panel that includes the genes affected, the variants, the consequence types, allelic frequencies in 1000 genomes (as a whole or in four groups with ancestors of different geographical origin: European, Asian, African and American) and ESP, as well as other parameters of interest. A known cause of generation of false positives and false negatives is the existence of regions with poor coverage or low quality in which variants are not reported in some of the individuals analyzed. Unlike any other tool, BiERapp manages efficiently these missing regions (if annotated in the VCF files) and consequently allows for variant filtering through pedigrees containing noisy or incomplete data.

When enough samples are available, complete family ‘pedigrees’ can be used and the experimental design is reasonable, the final number of candidates is usually a small figure and no more prioritization steps are necessary. In this case, the necessary subsequent experimental validation of only a small number of candidates contributes to the optimization of resources (time and budget) in the disease gene discovery process. However, this is not always the case. Often, the availability of other family members is not guaranteed or the own nature of the disease (rare diseases) precludes obtaining the necessary number of samples. Nevertheless, even in this case, the application of all the possible filters reduces in orders of magnitude the number of possible candidates. For example, most Mendelian diseases are caused by rare variants thus filtering out candidate variants present in control populations is of crucial importance (35).

If still a large number of candidate genes are obtained, other prioritization methods that make use of other gene properties (e.g. physical, genetic or functional relationships among them or to other known disease genes) can be used (36–38). As future improvement, we plan to provide a functional layer for further knowledge-based prioritization by connecting BiERapp to several methods implemented in our functional profiling tool Babelomics (39).

## SUPPLEMENTARY DATA

Supplementary Data are available at NAR Online.

Supplementary Data
