# Supplementary material for: A web-based interactive framework to assist in the prioritization of disease candidate genes in whole-exome sequencing studies
Source: Nucleic Acids Res. 2014 May 6;42(Web Server issue):W88–93. doi: 10.1093/nar/gku407 (PMC4086071; doi:10.1093/nar/gku407)
Supplement: Supplementary Data [file supp_42_W1_W88__index.html]

Supplementary Data 

# A web-based interactive framework to assist in the prioritization of disease candidate genes in whole-exome sequencing studies

## Supplementary Data

**Files in this Data Supplement:**

- Supplementary Data
