# Supplementary material for: A web-based interactive framework to assist in the prioritization of disease candidate genes in whole-exome sequencing studies
Source: Nucleic Acids Res. 2014 May 6;42(Web Server issue):W88–93. doi: 10.1093/nar/gku407 (PMC4086071; doi:10.1093/nar/gku407)
Supplement: Supplementary Data [file supp_gku407_nar-00503-web-b-2014-File003.docx]

**
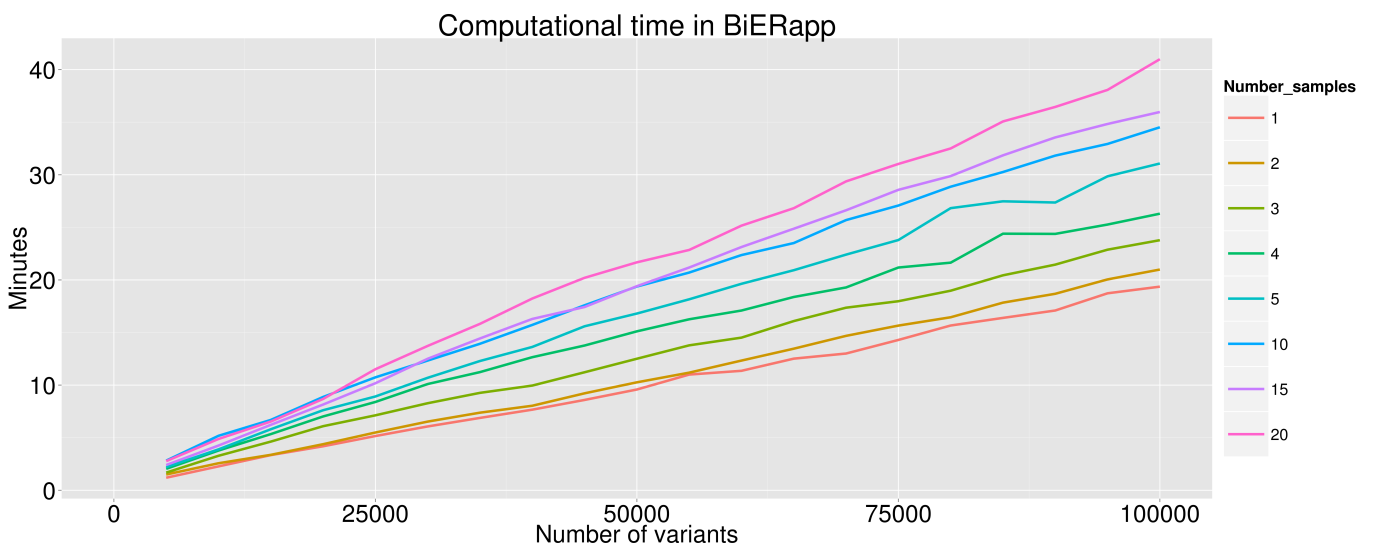
**

**Supplementary Figure 1.** Indexing runtimes in minutes for different number of exomes and variants.
